# Nurses’ refusals of patient involvement in their own palliative care

**DOI:** 10.1177/0969733020929062

**Published:** 2020-07-06

**Authors:** Stinne Glasdam, Charlotte Bredahl Jacobsen, Hanne Bess Boelsbjerg

**Affiliations:** 59568 Lund University, Sweden; 87049 University College Copenhagen, Denmark; 53154 Aarhus University, Denmark

**Keywords:** Patient involvement, nurse refusals, palliative care, power, thoughtlessness

## Abstract

**Background:**

Ideas of patient involvement are related to notions of self-determination and autonomy, which are not always in alignment with complex interactions and communication in clinical practice.

**Aim:**

To illuminate and discuss patient involvement in routine clinical care situations in nursing practice from an ethical perspective.

**Method:**

A case study based on an anthropological field study among patients with advanced cancer in Denmark.

**Ethical considerations:**

Followed the principles of the Helsinki Declaration.

**Findings:**

Two cases illustrated situations where nurses refused patient involvement in their own case.

**Discussion:**

Focus on two ethical issues, namely ‘including patients’ experiences in palliative nursing care’ and ‘relational distribution of power and knowledge’, inspired primarily by Hannah Arendt’s concept of *thoughtlessness* and a Foucauldian perspective on the medical clinic and power. The article discusses how patients’ palliative care needs and preferences, knowledge and statements become part of the less significant background of nursing practice, when nurses have a predefined agenda for acting with and involvement of patients. Both structurally conditioned ‘thoughtlessness’ of the nurses and distribution of power and knowledge between patients and nurses condition nurses to set the agenda and assess when and at what level it is relevant to take up patients’ invitations to involve them in their own case.

**Conclusion:**

The medical and institutional logic of the healthcare service sets the framework for the exchange between professional and patient, which has an embedded risk that ‘thoughtlessness’ appears among nurses. The consequences of neglecting the spontaneous nature of human action and refusing the invitations of the patients to be involved in their life situation call for ethical and practical reflection among nurses. The conditions for interaction with humans as unpredictable and variable challenge nurses’ ways of being ethically attentive to ensure that patients receive good palliative care, despite the structurally conditioned logic of healthcare.

## Introduction

Throughout the last 40 years, Western health policies, educational curricula and institutional health organisations have highlighted patients’ active participation in healthcare and are part of legal rights movements in many Western countries.^1,2^ Ideas of patient involvement are related to notions of self-determination and autonomy. However, patient involvement are often practised in decisions about treatment, healthy lifestyle and so on. Patient involvement are not always in alignment with complex interactions and communication in clinical practice. This article focuses on patient involvement in everyday situations in palliative nursing care practice arguing that attention to ethical issues is also important when it comes to small decisions and agenda setting of dialogues in routine care situations.

## Background

Patient involvement is an ideal in the Western healthcare system that stipulates that all people are treated as equal and autonomous individuals with the right and opportunity to determine their own lives when they are (potential) ill and enrolled in healthcare services. Philosophers and healthcare professionals have described patient involvement as an ethical imperative,^3,4^ which means an imperative resting on the principles of good clinical practice, respecting the aim that patients’ informed preferences should be the basis for professional actions.^3^

Concepts such as patient empowerment, patient involvement, shared decision-making and user-driven healthcare system are more or less synonymous with involving patients in their own healthcare situation.^1,5^ Angel and Frederiksen’s^6^ review of 33 articles shows that patient involvement is described as unquestionable and always advantageous to the patient. Both in medicine and nursing, patient participation in decisionmaking is articulated as a good method of involving patients in understanding their illness and managing symptoms.^6–8^ The notion includes the ideas that involving patients can lead to improved care outcomes,^9^ is a useful way to reduce costs in the healthcare system,^10,11^ enables patients to cope better with illness in everyday life,^12^ optimises the management of treatment and lifestyle^13^ and protects patient autonomy.^13–15^

Today’s concept of patient involvement can be regarded as a discursive meeting place for the interests and understandings of different actors. At this meeting place, the real meaning of the concept is negotiated and renegotiated, and a discussion takes place about whether given practices and methods are patientinvolving or not.^1,14^ Ideally, patient involvement happens through a negotiation of what is best to do in a concrete situation, where both healthcare professionals and patients bring knowledge and preferences into play, while healthcare professionals ensure that the professional and structural frameworks such as medical logic, economy and so on are respected.^3^ User manuals for the involvement of patients in clinical practice appear, in which healthcare service actors define common understandings and goals for patient-involving practices.^16,17^ However, these standardised assessments give a limited picture of how patient involvement is successfully achieved.^18,19^

Ideas of patient involvement are, as Madden and Speed^4^ argue, centred on a construction of the abstract, rational, compliant and self-managing patient or layperson. However, this construct may not always be in alignment with the complex interaction and communication taking place in clinical practice.^6^ In addition, there has been critique of the distribution of power and knowledge within the neoliberal organisational framework of modern healthcare systems, in which it seems unrealistic to expect to achieve patient involvement in its ideal form.^1,6,20,21^ The literature shows a tendency towards healthcare professionals understanding patient involvement as something offered to the patient.^6^ They often regard patient involvement as a question of giving patients free choices in different situations such as choosing treatment,^1,16^ choosing the order of tasks that professionals need to do^6,20^ and choosing changes in lifestyle.^6,22^ Studies show that patients often do not experience that they are involved in healthcare situations.^1,6,23^ Moreover, situations of patient involvement other than explicit free choice situations in clinical practice also call, ethically, for patient involvement. These situations include the opportunity to share thoughts about sorrow, suffering and other feelings with professionals,^19^ life history and life conditions of importance for health and healthcare with professionals,^23,24^ knowledge about one’s body, medical procedures and related experiences with professionals^23,25^ and so on. Such situations are of particular importance within palliative care, as the ideology of palliative care implicitly aims to relieve ‘the total pain’ of patients.^26^

Patient involvement in everyday situations in palliative nursing care practice is just as important when it comes to small decisions and agenda setting of dialogues in routine care situations as in decisions about treatment, healthy lifestyle and so on. However, these situations are easily overlooked. Based on two empirical cases, the article focuses on how nurses and patients with advanced cancer interact in hospitals, and illuminates situations where lack of patient involvement was visible. Thereby, this article aims to illuminate and discuss the involvement of patients in clinical routine care situations in nursing practice from an ethical perspective.

## Methods

This article is based on a medical anthropological study about palliative care needs among patients with advanced cancer in Denmark from 2010 to 2016. The study involved 16 patients and included interviews and participating observations with these individuals, their relatives and one individual who was appointed to be a professional caregiver. The focus of the study was on patients’ palliative care needs and their spiritual coping strategies when confronted with death.^27^

### Theoretical framework

This article uses two philosophers, Hanna Arendt^28,29^ and Michel Foucault,^30,31^ as inspiration to discuss ethics in relation to patient involvement in clinical routine care situations in nursing practice. Arendt^28^ finds that the essence of totalitarian government and the nature of every bureaucracy is to make functionaries and drivers in the administrative machinery out of people and in that way dehumanise them. The totalitarian government creates an internalised second opinion on our thoughts and actions and produces ‘thoughtlessness’, conditioned by the fact that most people are team-players and ‘play the game’.^28,29^ According to Schiff,^32^ Arendt’s concept of ‘thoughtlessness’ can be divided into three dimensions. First, ‘thoughtlessness’ can be a failure of conscience, that is, an individual failure to distinguish right from wrong, which may be fostered by institutional arrangements. Second, ‘thoughtlessness’ can be an ideological product of support and reproduction of systems of thoughts, actions and practices. Third, ‘thoughtlessness’ has an everyday dimension, where humans can be thoughtless to some extent as it is impossible to think about every little detail of everyday life.^32^ Although people are concerned about how ‘thoughtlessness’ blinds them to others’ suffering, they also practice ‘thoughtlessness’ within the other dimensions.^28,29,32^ The concept of ‘thoughtlessness’ seems relevant to reflect upon in healthcare settings.^33,34^

Foucault has a particularly critical eye for what characterises power in modern societies. He shows how actors in modern societies are caught in institutional structures, which pin down and form their actions.^30,31^ Foucault uses the term ‘medical gaze’ to denote the dehumanising medical separation of the patient’s body from the patient’s identity. Doctors modify and fit the patient’s story into a biomedical paradigm, filtering out non-biomedical issues.^30^ The medical gaze is assumed not only by dedicated doctors but by all professionals in the medical clinic. Foucault shows that the category ‘nurse’ appeared with the birth of the clinic and doctors’ need of assistance to observe patients in his absence.^31^ The clinic is a place of training and of the correlation of knowledge. Thus, the clinic consists of power relations and the constitution of a corpus of knowledge. Foucault shows how medicine creates an abusive power structure,^31^ where the medical gaze functions as a disciplinary technique.^30^ Power is omnipresent, relational and productive as it has the ability to bring things into action. As such, power will always generate some kind of resistance. Power is exercised throughout the social body, where it operates at micro levels of all social relationships, and is constituted through accepted forms of knowledge, scientific understanding and ‘truth’.^30,31^ Several researchers argue for the relevance of reflecting upon nursing practice inspired by Foucault’s thinking (e.g. Annerstedt and Glasdam^35^ and Clinton and Springer^36^).

### Recruitment

Patients with advanced cancer were recruited from medical care settings, consisting of one oncological ward, two palliative care units and one medical ward at hospitals, supplemented with recruitment from a cancer rehabilitation centre, and an organisation supporting patients with an ethnic minority background.^37^ All patients had palliative care needs. They gave the researchers consent to contact a relative and a professional such as a healthcare professional or chaplain, who were informed about the study either in person or by phone. Overall, 16 patients with cancer, 15 relatives and 15 healthcare professionals were included.

### Procedures of interviews and observations

The study consisted of both in-depth interviews and field observations.^38^ These were conducted on four hospitals wards and in the homes of the patients, located throughout Denmark. The mean time for following a person was 15 months but varied from 3 weeks to 6 years depending on the death of the person. The empirical materials consisted of handwritten observation notes and 64 interviews with patients, relatives and professionals. Interviews lasted between 24 and 170 min with an average of 75 min and were transcribed verbatim. Participants were interviewed twice, if their situation allowed. Interviews were supplemented with field observations containing elements from other patients’ hospitalisations and interactions with nurses. The field notes were kept in handwritten journals and on the computer.

### Analytical strategy

We have selected two of the total 16 cases based on the 16 participating patients in the study. These two cases were selected as they included examples of interactions between nurses and patients in healthcare settings, which demonstrated something about patient involvement. The selection was based on a desire to be able to discuss ethics in clinical routine care situations in nursing practice. That is, the two cases can be described as ‘telling cases’. They are not chosen because they are typical and necessarily similar to the other cases, but because they can help us spot something new, focussed on the exposure of new theoretical insights, and may help us expand our understanding of patient involvement in nursing.^39,40^ The case constructions focus on concrete interactions between nurses and patients, and quotes were selected from the empirical material to serve as illustration for the cases.

### Ethical considerations

The study followed the principles of the Helsinki Declaration.^41^ All participants gave written, informed consent before participation. The study obtained approval from the Danish Data Protection Agency (2013-41-1712). All the practice scenarios have been suitably anonymised to protect patient confidentiality.

## Findings

### Case 1: patient’s knowledge of her own body

Lisa was in her mid-60s. She had received experimental palliative treatment for a cancer. During many years of intermittent interventional chemotherapy, Lisa’s veins had transformed into scar tissue. This meant that it was difficult to take blood samples and insert peripheral venous catheters in Lisa’s veins. To evaluate the experimental cancer treatment, Lisa needed a scan, during which contrast fluid needed to be injected through a vein. She informed the nurse responsible for the injection about the difficulties related to her veins. The nurse answered that she would like to make an attempt before calling assistance from an experienced colleague.

Taking Lisa’s hand, the nurse assesses and selects a vein at the elbow flexion by applying a tourniquet. Lisa comments that her hand is cold: ‘I have fish blood’, she says…‘I have always said that because my hands tend to be a bit cold’. (Field notes)

Nurse disinfected Lisa’s arm and inserted the needle. It hurt. The cannula entered the vein, but destroyed it. The nurse tried to insert the needle in the other arm’s elbow flexion, but that also failed. She signalled to her colleague in the control room that she should call assistance from an anaesthetic nurse.

The nurse explains to Lisa: ‘I must always try to insert a cannula in the veins of the hands or feet before I call a colleague’. (Field notes)

The anaesthetic nurse arrived and tried in vain to insert the venous catheter at almost the same place. Lisa’s pain increased.

Suddenly, Lisa starts to get extremely tense. She seems afraid, as if she wants to leave her body. Everyone in the room reacts, as she is almost screaming ‘ouch!’. She looks like she is about to have a panic attack. (Field notes)

They all tried to comfort her. A nurse found a blanket for Lisa, as she had become cold. Lisa regained inner balance and calmness. The anaesthetic nurse asked if she could insert the venous catheter in the back of her hand. Lisa accepted. This time, the nurse succeeded and all present expressed relief. The nurse carefully positioned the cannula with several pieces of plaster. After the scan, the nurse apologised for all the attempts to insert the needle. Lisa responded:

It is surely not your fault. If you had done it on purpose, it would be different! As it is, there is nothing that can be done about it. (Field notes)

After the scan, Lisa should have had blood samples taken at another department (Clinical Biochemistry) by another nurse. She repeated the information about the condition of her veins to the nurse. The nurse knocked on Lisa’s veins and decided to insert a cannula in her elbow flexion after removing one of the plasters. It hurt, and Lisa moaned loudly in pain. Lisa hunched her shoulders far up towards her ears. She became tense again.

The nurse gets very surprised that it is so painful, saying: ‘Usually, the elbow flexion is the least sensitive place on the arm’. (Field notes)

The nurse interrupted the insertion and said she would find another place and apply what she called a ‘baby needle’. This meant that the actual blood collection would take longer than usual, but the plug would be less painful. Then, she knocked on the tiny vein that ran in the wrist and asked, if it was okay to insert the needle there. Lisa agreed. The nurse took one of the small needles and inserted it. Then, the blood flowed slowly and successfully.

### Case 2: patient’s concern about her impending death

Sarah was a well-functioning woman in her mid-70s. She had lung cancer and had been admitted several times to a palliative medical unit due to respiratory distress. She smoked, even though she often needed oxygen. During the hospitalisation, she included others in her thoughts on her impending death. For example, Sarah told a nurse: ‘I am tired of living’. Her husband had died years previously of cancer, and her son was also dead. She continued: ‘When I’m dead, I’ve arranged for a man to pick up and sell my furniture. The money should go to charity’. The nurse interrupted and said: ‘It is lovely weather today. Want to sit in the garden to have breakfast?’ Sarah replied: ‘It might be a little early, but I would like to go into the garden later on, when I need to smoke’.

Sarah went in the garden for lunch. The nurse and a fellow patient were present when Sarah said: ‘I am not afraid of dying!’. She continued explaining how she had mastered the practicality of her impending death. She did not expect anyone whom she knew to show up for her funeral. Her social network was very limited. However, she had arranged payment for her coffin, written down that she should be cremated and where the urn should be placed. Her fellow patient burst out: ‘Please stop!’, and explained that she was not ready for this kind of conversation, so their talk shifted into another direction.

Despite Sarah’s clarity on practical matters related to her forthcoming death, there were several episodes during the field study that pointed to the fact that there was also something that worried Sarah and aroused her anxiety. In those situations, she often invited the nurses to engage with her worries.

Scene 1: Sarah’s respiratory distress required her to have personal hygiene assistance. The nurse was in Sarah’s room helping prepare Sarah to take a shower. The nurse asked Sarah to put her legs over the edge of the bed so she could get out of bed and enter the bathroom. Sitting on the edge of the bed, Sarah suddenly exclaimed: ‘I’m looking forward to when “He” is coming and picking me up’. The nurse was standing close to Sarah, waiting for Sarah to take off the oxygen mask and get ready to get out of bed. The nurse replied, ‘I can understand that’ and continued in a teasing tone: ‘Is it so bad to be here?’. Sarah replied: ‘No! It is not at all. After all, everyone is sweet and kind to me’. Sarah felt rebuked, left the subject, stepped down from the bed and the nurse supported her into the bathroom.

Scene 2: As shifts changed, the night watch reported to the following day watch that Sarah had had problems with anxiety in the morning:

Sarah was already awake at six o’clock and was confused. She had a violent nightmare and was completely out of herself. She was offered some sedative medication so she could fall asleep again, but Sarah didn’t want it. She was helped out of bed and had a cup of coffee. Then, she calmed down again.

The nurse who took over responsibility for Sarah in day-care received the handover and wrote a note in the journal. Shortly thereafter, the nurse went to Sarah with her morning medication: ‘Good morning. Well, how are you, Sarah?’. Sarah immediately began to tell that she had not slept well because of a terrible nightmare. The nurse replied: ‘Well, that was not so nice’, and then she said to Sarah: ‘Now, you just have to think about something else, and then you will be fine again’. The nurse left Sarah’s room and continued to distribute medication to the other patients on the ward.

## Discussion

The discussion will focus on two ethical issues in the cases, namely ‘involvement of patients’ ‘experiences in palliative nursing care’ and ‘relational distribution of power and knowledge’, inspired by Arendt’s concept of ‘thoughtlessness’^28,29^ and a Foucauldian perspective on the medical clinic and related power.^30,31^

### Involvement of patients’ experiences in palliative nursing care

The interactions between patients and healthcare professionals begin with the fact that a person experiences something wrong with her or his own body and therefore contacts the healthcare system for help. Initially, it is the patient (or a relative) who involves healthcare professionals in her or his life as a result of which a relationship between the patient and healthcare professionals arises.^42^ In this process, patients partly hand over the responsibility for their bodies to healthcare professionals. This means that healthcare professionals, who have little direct access to patients’ bodily and emotional experiences, end up with a primary responsibility for determining *how* and *how much* patients’ expressions about bodily and emotional issues are to be emphasised in specific situations. Ideologically, this calls for professional ethical attention in palliative care.^26^ However, such professional attention seems absent in both *cases 1* and *2*. In *case 1*, Lisa actively offers knowledge of her body to facilitate the nurse’s work and minimise her own physical discomfort and anxiety. Lisa shares clinically relevant knowledge with the nurses about her body and its invisible dysfunction, but they do not listen to her. In *case 2*, on numerous occasions, Sarah invites nurses to a dialogue about her imminent death, which nurses refuse. This is remarkable since palliative care rests on ideals that include end-of-life dialogue to relieve patient suffering.^26^ Inspired by Arendt,^28^ Roberts and Ion^43^ discuss how such behaviour can be regarded as ‘thoughtlessness’ on the part of the nurses who stick to an ‘unreflective strategy’ in which nurses do not think about consequences outside the structural framework of healthcare. They are subject to the structural framework of healthcare^30^ with reproduction of the systems of thoughts, actions and practices.^32^ Nurses have the power to define when and how involvement of patients is going to happen in clinical routine care situations in nursing practice. Thus, nurses do as implicitly expected, as they are socialised in a culture that *per se* does not involve patients’ perspectives^44^ and favours an instrumental rule-governed rationality. Patients’ attempts to set an agenda or contribute knowledge to nurses may unconsciously be rejected because of ‘thoughtlessness’ conditioned by nurses’ habitual understanding that this is time-consuming and/or irrelevant to core clinical tasks.^3^ The actions of nurses are embedded in the structural, bureaucratic conditions in which nurses often unconsciously work in a way that confirms the inherent logic and reward systems of the system.^44^ As such, the cases illustrate how institutional arrangements in healthcare with its historical roots and culture, as well as the power relation between patient and nurses, are fostering nurses’ ‘thoughtlessness’ as a failure of conscience and/or an ideology serving the effectiveness of a system. In addition, the individual nurse may also have an everyday occurrence of ‘thoughtlessness’ due to a lack of attention.^32^

Another possible explanation is to be found in the unpredictable nature of patients’ actions and initiatives. ‘Thoughtlessness’ is closely related to the ignorance of the plurality and unpredictability of the human condition. According to Arendt,^29^ it is part of the human condition that people are different. At the same time, it is an international ethical norm that people should be given equal rights and status, regardless of their social positions and different needs.^45^ When representatives of a system try to gain control by not allowing or acknowledging the plurality and unpredictability of the human condition, a tendency towards totalitarianism evolves.^29^ In *cases 1* and *2*, ‘thoughtlessness’ can be interpreted as the outcome of an authoritarian regime in which the medical logic dominates, ruled by values of effectiveness and cost-reductions.^34^ In this regime, nurses keep the focus on solving the system’s tasks without, in principle, taking into account plurality and unpredictability among patients.

Arendt^29^ defines action as a higher form of human interaction, always involving the unforeseen, appearing spontaneously and in relation to others.^34^ Regarding the expressions of the patients in *cases 1* and *2* as moments of actions, it can be the unpredictability, or the open outcome, that make these actions difficult for – or unpopular with – the nurses. Often, people prefer to retain a grip on reality and avoid what is unforeseen.^29^ Nurses’ way of handling unpredictability in routine clinical care situations in nursing practice includes the possibility and risk of performing harmful deeds.^28,43^ This is due to individual failure in distinguishing right from wrong in the situation and/or as a consequence of nurses’ socialisation into the medical ideology of the healthcare system.^31,32,44^ Kohlen^34^ argues in continuation of Arendt that whoever tries to remove unpredictability from action will destroy what is defining us as human. To avoid dehumanisation in healthcare, it is necessary to allow contingencies and unplanned aspects in specific meetings with patients. In other words, nurses must free themselves from instrumental and technical approaches in order to encompass and accept patients’ unpredictable initiatives in the form of invitations to become involved in their (life) situations.^34^

### Relational distribution of power and knowledge

The practice of ‘thoughtlessness’ must be understood – and ethically challenged – in relation to the medical clinic.^43^ Nurses are socialised into the medical clinic with its distinctive logic and language.^28,30,44,46^ The medical context in which meetings between patients and nurses occur is governed by a medical logic where physical needs and treatments have the highest priority.^47^ Foucault^30^ shows how the ‘medical gaze’ gives power to the specific knowledge gathered by distancing the medical professionals from the patients by circumscribing them into medical conditions. According to Foucault, the power to acknowledge what is considered as ‘relevant knowledge’ rules the interaction of the partners involved.^30^ *Case 1* illustrates how the patient’s knowledge of her veins and their nature is not recognised as useful knowledge for nurses in their priorities and choices when inserting venous cannula and taking blood samples. Patients’ knowledge *per se* does not enable them to participate in joint decisions with healthcare professionals, as other studies have shown.^1,48^ Patients need to be assigned power by nurses in their interactions with patients, if patients’ knowledge is to be applied in clinical practice. Most patients adjust to a subordinate position in the healthcare system, and try to act in relation to a notion of being ‘a good patient’. Campbell and colleagues show that characteristics of a good patient include obedience, patience, politeness, listening, enthusiasm for treatment, intelligence, physical cleanliness, honesty, gratitude and lifestyle adaptations.^49^ These qualities might hamper the patients’ opportunities to initiate a relationship that facilitates knowledge sharing. An additional factor, which enhances this challenge, is that empirical observations show that patients interpret the behaviour of healthcare professionals and what is expected of them, as well as the structural framework to which they are subordinated.^48^ This interpretation of patients’ behaviour can be seen in both Lisa in *case 1* and Sarah in *case 2*. In *case 1*, Lisa draws attention to her knowledge about her body, but the nurses do not offer a relational response that includes her experiences. Through silence, Lisa accepts her subordinated position and the nurses’ decisions. Only when Lisa’s expression cannot be overheard, the nurses listen to her and change their strategy. In *case 2*, Sarah leaves the theme of her impending death as nurses change the subject.

The nurses meet the moment of action when the patient indicates their wish to be involved in two different ways in the cases. In *case 1*, the nurses respond to Lisa by ignoring her knowledge about her own body and by being insensitive to her underlying anxiety of not knowing the outcome of the experimental treatment. This ignorance is partly produced out of neglect of the information offered by Lisa and partly as a result of the organisational procedures for assistance which nurses need to follow in the clinic. In *case 2*, the nurses also meet Sarah and her invitations to talk about her impending death by either ignoring her existential concerns or neglecting them by the use of humour. The use of humour can be regarded as an exercise of power,^31^ setting the agenda for problems that can be talked about and not talked about in the nurse–patient meeting, with a risk of exacerbating the patient’s existential needs. The nurses’ use of humour as a mechanism to ignore a topic, raised by a patient, is not good ethical nursing.^26,44^ It is also in direct contrast to International Council of Nurses’ (ICN)^45^ intention that nurses have an obligation to respect people’s health rights at all times and in all places, including the way they express their needs, worries or wishes. By ignoring patients’ expressions of concern, the nurses undermine the fundamentals of patient involvement and show a lack of understanding of the human condition. Using humour to ignore significant concerns allows nurses to maintain their planning and prioritising actions on the part of patients by averting the contingency and invitation to get involved in patients’ situations. Humour can be a powerful tool for warding off or converting an undesirable situation into a desired, controllable situation, while also indirectly ridiculing patients’ priorities or laughing at their human frailties behind a compassionate outward expression.^50^ Nurses’ strategies of distraction, ignorance or neglect by the use of humour illustrate how involvement of patients’ experiences depends on acknowledgement of the issues they raise by nurses. In continuation of the thinking of Arendt,^28^ the modern healthcare system of today calls for critical, ethical reflection as it carries a great risk of increasingly promoting and rewarding nurses who dehumanise patients, as the system’s structural framework shapes nurses to be part of a form of totalitarianism where ‘thoughtlessness’ is included as an embedded condition in nursing. The article’s findings demonstrate the necessity for researchers to assume an obligation to explore poor clinical practice. Furthermore, it points to the necessity for nurses to learn from poor practice that may distress or harm patients, and to increase their awareness of their own ‘thoughtlessness’.

Studies show that patients quickly detect authoritarian ways of communicating and may interpret it to mean that their knowledge, questions and preferences do not have space and priority in their interactions with healthcare professionals.^48,51^ In the medical clinic, the order of power and knowledge is staggered, and healthcare professionals are positioned over patients in terms of the patient’s disease, care and treatment.^30^ Grimen^52^ describes this distribution of power and knowledge in the modern healthcare system as a closely linked connection between power, risk and trust. When patients enrol in the healthcare system, they put their trust in the insights and intentions of healthcare professionals, and in that way assign power to healthcare professionals. Specifically, they assign power to act on their bodies, power to assess what is possible to do and power to assess what is too risky to do.^52^ Ultimately, this means that patients give healthcare professionals the responsibility for acting on their bodies during an admission. In other words, patients entrust part of their self-determination and autonomy to healthcare professionals who, under strict regulations in terms of their authorisations, exercise professional autonomy.^53^ Historically, professionals have been granted the right to make decisions about patients’ bodies, despite the fact that there have been many actions over the past 40 years to change this trajectory.^1,2^ A part of the explanation for this historical process could be that all actors more or less accept and respect the order of the clinic with its exclusion of patients and their bodily knowledge and expressions, besides the basic economic, political, social and symbolic positions of power. Another part of the explanation is that both patients and professionals are locked into a self-confirming and self-enhancing cycle of homologies, which form the order of the clinic and lead to situations where this order is seldom discussed by or among the actors.^54^ A historical process of change demands that professionals re-assign the right to make decisions about the patient’s body to the patient, not just in words but also in actions in clinical practice. History is transformed into culture and cultural arbitrariness is transformed into naturalness, understood as self-evident. It is an ethical challenge to make ‘small’ decisions around routine care practices visible in order to regain patient involvement in its arbitrary, non-statutory character and its socio-logical necessity.^54^ From an ethical perspective, history must return to its starting point, namely that the patient’s life and disease situation returns to the centre of the relationship between professionals and patients. Studies show that it is difficult for nurses to meet their own humanistic ideals and relate ethically to the patient’s needs and suffering when the medical logic and the neoliberal organisational framework are prevalent and only unplanned time slots allow nurses to prioritise psychosocial needs for patients and relatives.^44,46^ The acknowledgement of the importance of relating to patients’ experiences as valid knowledge would support an ethically sound nursing practice that does not belittle the existential dimension of being ill and dependent on provision of care.

Finally, the article’s inspiration, from chosen concepts of Foucault’s and Arendt’s thinking, offers fruitful ways to analyse and discuss dehumanisation in healthcare by focussing on ways in which unpredictability and uncertainty in patient–nurse situations are managed in relation to patient involvement. The two philosophers have different starting points and ways of thinking, but both argue that, on the one hand, people’s opportunities and thought patterns are conditional on the structural framework of the bureaucracy, including the healthcare system. On the other hand, people have a tendency to accept and thereby support the conditions and systems they have in their lives.^55,56^ Furthermore, Arendt and Foucault show that people created historical conditions and so conditions can also be changed by people, although there is a built-in sluggishness in changing historical conditions, and associated knowledge, action and thought systems. The perspectives of Arendt and Foucault point to the urgent need for a critical approach to current nursing practice, help us understand these challenges and inspire us in facing them.^55,57^ Overall, the study raise an ethical question for the researchers. It is an ethical demand to find a balance between when a researcher should study the actual circumstances exclusively to get to know them better, and when a researcher should intervene when what they find can be classified as practice that distresses and harms patients. It has to be reflected upon at which point researchers ought to mention poor practice to nurses and managers in a given ward, and when researchers ought to incite a patient or their relative to complain about the care that has been given.

## Conclusion

There are limitations and challenges in involving patients in clinical nursing practice, partly because of the ‘thoughtlessness’ of nurses, and partly due to the distribution of power and knowledge in healthcare. The power to define the content of conversations and the relational aspects of the interactions is rarely negotiated by the patient, but often set by the nurse. The medical and institutional logic of the healthcare service often sets the framework for the exchange between professional and patient and creates ‘thoughtlessness’ among nurses. This kind of interaction ignores what Arendt has pointed out as the human condition. By not taking the plurality of humankind and the unpredictability of what actions might lead to into account, patient involvement has limited options in nursing everyday practice, as it *per se* is not included in the healthcare structural framework. The patient’s needs, preferences, knowledge and statements stay in the background as less significant for nursing practice, when nurses have a predefined agenda for the way they act with and involve patients. Consequently, the nurses’ fixed agenda becomes the core of the conversation and interaction, while patients’ inputs become peripheral, and the possibility of acting responsively to the human condition is neglected. Moreover, when professional, academic and political frameworks in into healthcare services decide and determine the distribution of knowledge and definition of significant knowledge, this may overrule relational aspects of knowledge-sharing and most importantly the insight the subjective issues at stake. These conditions challenge nurses’ ideas of being ethical attentive to ensure that patients receive good palliative care. Nurses need to become reflective and discover the tendency towards ‘thoughtlessness’ and power relations if patient involvement is to be taken seriously and dehumanisation of patients in palliative care avoided.
